# 1,3-Bis(4-chloro­phen­yl)-1-methyl-1*H*-benzo[*f*]chromene

**DOI:** 10.1107/S1600536810022610

**Published:** 2010-06-23

**Authors:** Rener Chen, Qizhong Zhou

**Affiliations:** a School of Pharmaceutical and Chemical Engineering, Taizhou University, Taizhou 317000, People’s Republic of China

## Abstract

The title compound, C_26_H_18_Cl_2_O, is a heterocyclic structure consisting of a benzo[*f*]chromene ring and two aromatic rings. The non-H atoms of the benzo[*f*]chromene ring are almost coplanar (rms deviation = 0.107 Å), and the methyl C atom lies 1.340 (4) Å from the mean plane of the benzo[*f*]chromene ring. The chromene ring forms dihedral angles of 88.45 (2)° with the benzene ring linked to the quaternary C atom and 50.74 (3)° with the benzene ring linked to the 3-position, while the dihedral angle between the two benzene rings is 67.58 (3)°.

## Related literature

For the construction of a 4*H*-chromene scaffold, see: Shi & Shi (2007 ▶); Zeni & Larrock (2004 ▶). For the biological and pharmacological activity of 4*H*-chromene derivatives, see: Kidwai *et al.* (2005 ▶). For 4*H*-chromene derivatives possessing a tertiary carbon in the scaffold, see: Liu *et al.* (2010 ▶); Wang & Zhu (2010 ▶). For iron trichloride-catalysed synthesis of 4*H*-chromenes, see: Kozlikovskii *et al.* (1986 ▶).
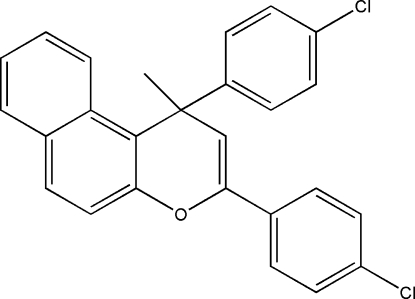


## Experimental

### 

#### Crystal data

C_26_H_18_Cl_2_O
*M* *_r_* = 417.33
Triclinic, 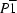

*a* = 8.9554 (6) Å
*b* = 9.4439 (6) Å
*c* = 12.3206 (7) Å
α = 93.511 (2)°
β = 102.0453 (15)°
γ = 94.287 (2)°
*V* = 1013.05 (11) Å^3^
*Z* = 2
Mo *K*α radiation
μ = 0.34 mm^−1^
*T* = 296 K
0.42 × 0.37 × 0.28 mm

#### Data collection

Rigaku R-AXIS RAPID diffractometer
Absorption correction: multi-scan (*ABSCOR*; Higashi, 1995 ▶) *T*_min_ = 0.874, *T*_max_ = 0.910
10050 measured reflections
4596 independent reflections
2783 reflections with *F* ^2^ > 2σ(*F* ^2^)
*R*_int_ = 0.021

#### Refinement

*R*[*F* ^2^ > 2σ(*F* ^2^)] = 0.042
*wR*(*F* ^2^) = 0.095
*S* = 1.00
4596 reflections
264 parameters
H-atom parameters constrained
Δρ_max_ = 0.28 e Å^−3^
Δρ_min_ = −0.33 e Å^−3^

### 

Data collection: *PROCESS-AUTO* (Rigaku, 1998 ▶); cell refinement: *PROCESS-AUTO*; data reduction: *CrystalStructure* (Rigaku/MSC, 2004 ▶); program(s) used to solve structure: *SHELXS97* (Sheldrick, 2008 ▶); program(s) used to refine structure: *SHELXL97* (Sheldrick, 2008 ▶); molecular graphics: *ORTEP-3 for Windows* (Farrugia, 1997 ▶); software used to prepare material for publication: *CrystalStructure* (Rigaku/MSC, 2004 ▶).

## Supplementary Material

Crystal structure: contains datablocks global, I. DOI: 10.1107/S1600536810022610/zq2041sup1.cif

Structure factors: contains datablocks I. DOI: 10.1107/S1600536810022610/zq2041Isup2.hkl

Additional supplementary materials:  crystallographic information; 3D view; checkCIF report

## Figures and Tables

**Fig. 1.**
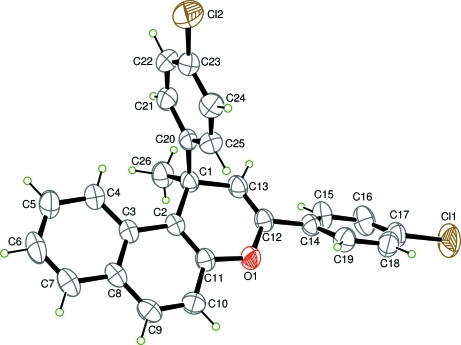
The molecular structure of the title compound, with the atomic labeling scheme. Displacement ellipsoids are drawn at the 50% probability level.

**Fig. 2.**
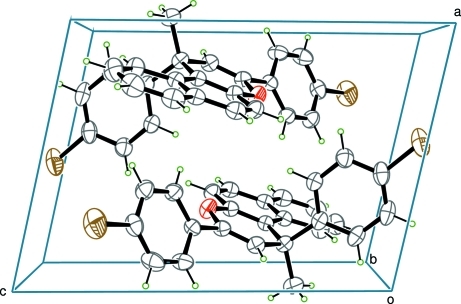
The packing of the crystal structure of the title compound. The C, O and Cl atoms are grey, red and brown, respectively.

## supplementary crystallographic information

### Comment

Chromenes (2*H*- and 4*H*-chromenes) constitute an important class of scaffolds found in many naturally occurring and synthetic molecules showing unique biological and pharmacological activities including spasmolytic, diuretic, anticoagulant, anticancer activities and so on. In this article, the crystal structure of the title compoud 1,3-di(4-chloro-phenyl)-1-methyl-1*H*-benzo[*f*]chromene was determined (Fig. 1–2). Its main structure unit consists of a benzo[*f*]chromene ring and two aromatic rings, in which the non-H atoms of the benzo[*f*]chromene ring are almost coplanar, and the methyl atom C26 lies 1.340 (4) Å from the benzo[*f*]chromene ring. The chromene ring forms dihedral angles of 88.45 (2)° with the benzene ring linked to the quaternary carbon atom C1, and 50.74 (3)° with the other benzene ring linked to C12, while the dihedral angle between the two benzene rings is 67.58 (3)°.

### Experimental

The title compound was synthesized by treating (*E*)-1,3-bis(4-chlorophenyl)but-2-en-1-one (0.291 g,1 mmol) with 2-naphthol (0.144 g,1 mmol) in the presence of ferric trichloride (0.16 g, 1.0 mmol) as a catalyst in 1,2-dichloroethane (3 ml) under stirring at reflux for 6 h. Upon completion, the resulting mixture was diluted with dichloromethane (10 ml) and filtered through Celite. After evaporation of the solvent under vacuum, the residue was purified by column chromatography on silica gel (200–300 mesh) using Petroleum ether-EtOAc (30:1) as eluent to give the title compound (0.19 g, 40%). Suitable crystals of the title compound were obtained by slow evaporation of ethanol solution at room temperature.

### Refinement

H atoms were placed in calculated position with C—H = 0.96 Å (*sp*), C—H = 0.93 Å (*sp^2^*), C—H = 0.93 Å (aromatic H atoms). All H atoms are included in the final cycles of refinement using a riding mode, with *U*_iso_(H) = 1.2*U*_eq_ of the carrier atoms.

### Crystal data

**Table:**

C_26_H_18_Cl_2_O	*Z* = 2
*M**_r_* = 417.33	*F*(000) = 432.00
Triclinic, *P*1	*D*_x_ = 1.368 Mg m^−^^3^
Hall symbol: -P 1	Mo *K*α radiation, λ = 0.71075 Å
*a* = 8.9554 (6) Å	Cell parameters from 6736 reflections
*b* = 9.4439 (6) Å	θ = 3.0–27.4°
*c* = 12.3206 (7) Å	µ = 0.34 mm^−^^1^
α = 93.511 (2)°	*T* = 296 K
β = 102.0453 (15)°	Chunk, colorless
γ = 94.287 (2)°	0.42 × 0.37 × 0.28 mm
*V* = 1013.05 (11) Å^3^	

### Data collection

**Table:**

Rigaku R-AXIS RAPID diffractometer	2783 reflections with *F*^2^ > 2σ(*F*^2^)
Detector resolution: 10.00 pixels mm^-1^	*R*_int_ = 0.021
ω scans	θ_max_ = 27.5°
Absorption correction: multi-scan (*ABSCOR*; Higashi, 1995)	*h* = −11→11
*T*_min_ = 0.874, *T*_max_ = 0.910	*k* = −12→12
10050 measured reflections	*l* = −15→14
4596 independent reflections	

### Refinement

**Table:**

Refinement on *F*^2^	*w* = 1/[σ^2^(*F*_o_^2^) + (0.001*P*)^2^ + 0.920*P*] where *P* = (*F*_o_^2^ + 2*F*_c_^2^)/3
*R*[*F*^2^ > 2σ(*F*^2^)] = 0.042	(Δ/σ)_max_ < 0.001
*wR*(*F*^2^) = 0.095	Δρ_max_ = 0.28 e Å^−^^3^
*S* = 1.00	Δρ_min_ = −0.33 e Å^−^^3^
4596 reflections	Extinction correction: *SHELXL97* (Sheldrick, 2008)
264 parameters	Extinction coefficient: 0.0188 (6)
H-atom parameters constrained	

### Special details

**Table:**

Refinement. Refinement using reflections with *F*^2^ > 2.0 σ(*F*^2^). The weighted *R*-factor(*wR*), goodness of fit (*S*) and *R*-factor (gt) are based on *F*, with *F* set to zero for negative *F*. The threshold expression of *F*^2^ > 2.0 σ(*F*^2^) is used only for calculating *R*-factor (gt).

### Fractional atomic coordinates and isotropic or equivalent isotropic displacement parameters (Å^2^)

**Table:**

	*x*	*y*	*z*	*U*_iso_*/*U*_eq_	
Cl1	0.25919 (11)	−0.05748 (9)	0.82025 (6)	0.0899 (3)	
Cl2	0.54898 (11)	0.29006 (9)	−0.01095 (6)	0.0875 (2)	
O1	0.27277 (19)	0.50754 (14)	0.53308 (12)	0.0492 (4)	
C1	0.1329 (2)	0.4692 (2)	0.29332 (18)	0.0405 (5)	
C2	0.2049 (2)	0.6121 (2)	0.35548 (17)	0.0380 (4)	
C3	0.2145 (2)	0.7416 (2)	0.29947 (18)	0.0406 (5)	
C4	0.1688 (2)	0.7468 (2)	0.1828 (2)	0.0521 (6)	
C5	0.1790 (3)	0.8718 (2)	0.1324 (2)	0.0614 (7)	
C6	0.2364 (3)	0.9992 (2)	0.1961 (2)	0.0635 (7)	
C7	0.2840 (2)	0.9991 (2)	0.3080 (2)	0.0543 (6)	
C8	0.2758 (2)	0.8720 (2)	0.3627 (2)	0.0430 (5)	
C9	0.3277 (2)	0.8730 (2)	0.4787 (2)	0.0473 (5)	
C10	0.3245 (2)	0.7510 (2)	0.53063 (19)	0.0465 (5)	
C11	0.2644 (2)	0.6220 (2)	0.46756 (18)	0.0403 (4)	
C12	0.2041 (2)	0.3771 (2)	0.48333 (18)	0.0406 (5)	
C13	0.1397 (2)	0.3577 (2)	0.37734 (19)	0.0431 (5)	
C14	0.2173 (2)	0.2685 (2)	0.56609 (18)	0.0401 (4)	
C15	0.0923 (2)	0.1769 (2)	0.5723 (2)	0.0542 (6)	
C16	0.1045 (2)	0.0765 (2)	0.6511 (2)	0.0609 (7)	
C17	0.2428 (3)	0.0687 (2)	0.7219 (2)	0.0532 (6)	
C18	0.3693 (2)	0.1574 (2)	0.7168 (2)	0.0538 (6)	
C19	0.3551 (2)	0.2575 (2)	0.63937 (19)	0.0483 (5)	
C20	0.2310 (2)	0.4205 (2)	0.21085 (17)	0.0419 (5)	
C21	0.1736 (3)	0.3746 (2)	0.09965 (19)	0.0542 (6)	
C22	0.2691 (3)	0.3322 (2)	0.0309 (2)	0.0618 (7)	
C23	0.4242 (3)	0.3367 (2)	0.0735 (2)	0.0571 (6)	
C24	0.4842 (2)	0.3788 (2)	0.1839 (2)	0.0547 (6)	
C25	0.3879 (2)	0.4192 (2)	0.25085 (19)	0.0475 (5)	
C26	−0.0378 (2)	0.4756 (2)	0.2390 (2)	0.0551 (6)	
H4	0.1309	0.6630	0.1389	0.063*	
H5	0.1474	0.8717	0.0556	0.074*	
H6	0.2420	1.0839	0.1619	0.076*	
H7	0.3229	1.0842	0.3498	0.065*	
H9	0.3648	0.9586	0.5202	0.057*	
H10	0.3615	0.7523	0.6071	0.056*	
H13	0.0939	0.2670	0.3510	0.052*	
H15	−0.0010	0.1823	0.5234	0.065*	
H16	0.0198	0.0156	0.6555	0.073*	
H18	0.4629	0.1501	0.7649	0.065*	
H19	0.4400	0.3190	0.6363	0.058*	
H21	0.0688	0.3724	0.0707	0.065*	
H22	0.2287	0.3010	−0.0432	0.074*	
H24	0.5889	0.3798	0.2127	0.066*	
H25	0.4289	0.4468	0.3256	0.057*	
H261	−0.0469	0.5428	0.1831	0.066*	
H262	−0.0806	0.3831	0.2052	0.066*	
H263	−0.0921	0.5047	0.2948	0.066*	

### Atomic displacement parameters (Å^2^)

**Table:**

	*U*^11^	*U*^22^	*U*^33^	*U*^12^	*U*^13^	*U*^23^
Cl1	0.1224 (7)	0.0753 (5)	0.0833 (5)	0.0203 (4)	0.0317 (5)	0.0476 (4)
Cl2	0.1234 (7)	0.0946 (6)	0.0600 (4)	0.0380 (5)	0.0429 (4)	0.0140 (4)
O1	0.0640 (10)	0.0346 (8)	0.0440 (8)	−0.0043 (7)	0.0019 (7)	0.0087 (6)
C1	0.0442 (12)	0.0305 (10)	0.0439 (11)	0.0003 (9)	0.0033 (9)	0.0046 (8)
C2	0.0383 (11)	0.0323 (11)	0.0446 (11)	0.0043 (9)	0.0101 (9)	0.0065 (9)
C3	0.0414 (11)	0.0340 (11)	0.0490 (12)	0.0066 (9)	0.0127 (10)	0.0096 (9)
C4	0.0627 (15)	0.0421 (13)	0.0529 (14)	0.0086 (11)	0.0117 (12)	0.0126 (10)
C5	0.0797 (19)	0.0519 (15)	0.0579 (15)	0.0144 (13)	0.0186 (14)	0.0216 (12)
C6	0.0733 (18)	0.0409 (14)	0.085 (2)	0.0116 (13)	0.0274 (15)	0.0283 (13)
C7	0.0544 (14)	0.0338 (12)	0.0771 (18)	0.0028 (10)	0.0179 (13)	0.0117 (11)
C8	0.0401 (11)	0.0315 (11)	0.0598 (14)	0.0037 (9)	0.0150 (10)	0.0062 (9)
C9	0.0452 (13)	0.0346 (11)	0.0615 (14)	−0.0016 (9)	0.0131 (11)	−0.0011 (10)
C10	0.0487 (13)	0.0407 (12)	0.0469 (12)	−0.0022 (10)	0.0058 (10)	−0.0006 (10)
C11	0.0410 (11)	0.0336 (11)	0.0466 (12)	0.0017 (9)	0.0095 (10)	0.0083 (9)
C12	0.0391 (11)	0.0332 (11)	0.0493 (12)	0.0003 (9)	0.0083 (10)	0.0086 (9)
C13	0.0465 (12)	0.0311 (11)	0.0502 (13)	−0.0014 (9)	0.0075 (10)	0.0070 (9)
C14	0.0425 (12)	0.0356 (11)	0.0448 (11)	0.0046 (9)	0.0135 (10)	0.0080 (9)
C15	0.0407 (13)	0.0519 (14)	0.0718 (16)	0.0053 (11)	0.0108 (12)	0.0225 (12)
C16	0.0542 (15)	0.0499 (15)	0.0861 (19)	0.0028 (11)	0.0262 (14)	0.0271 (13)
C17	0.0697 (17)	0.0452 (13)	0.0521 (14)	0.0150 (12)	0.0220 (13)	0.0196 (11)
C18	0.0573 (15)	0.0542 (15)	0.0485 (13)	0.0079 (12)	0.0046 (11)	0.0126 (11)
C19	0.0467 (13)	0.0471 (13)	0.0496 (13)	−0.0023 (10)	0.0076 (11)	0.0084 (10)
C20	0.0541 (13)	0.0300 (10)	0.0390 (11)	0.0037 (9)	0.0031 (10)	0.0066 (8)
C21	0.0660 (16)	0.0473 (14)	0.0425 (12)	0.0097 (12)	−0.0054 (11)	0.0039 (10)
C22	0.093 (2)	0.0555 (16)	0.0343 (12)	0.0178 (14)	0.0038 (13)	0.0045 (10)
C23	0.087 (2)	0.0459 (14)	0.0441 (13)	0.0187 (13)	0.0209 (13)	0.0105 (10)
C24	0.0596 (15)	0.0562 (15)	0.0498 (14)	0.0118 (12)	0.0125 (12)	0.0057 (11)
C25	0.0542 (14)	0.0457 (13)	0.0403 (11)	0.0046 (11)	0.0058 (10)	0.0004 (9)
C26	0.0474 (14)	0.0512 (14)	0.0624 (15)	0.0012 (11)	0.0015 (11)	0.0098 (11)

### Geometric parameters (Å, °)

**Table:**

Cl1—C17	1.743 (2)	C18—C19	1.378 (3)
Cl2—C23	1.740 (3)	C20—C21	1.389 (2)
O1—C11	1.385 (2)	C20—C25	1.389 (3)
O1—C12	1.381 (2)	C21—C22	1.386 (4)
C1—C2	1.536 (2)	C22—C23	1.375 (4)
C1—C13	1.517 (3)	C23—C24	1.375 (3)
C1—C20	1.547 (3)	C24—C25	1.371 (3)
C1—C26	1.542 (3)	C4—H4	0.930
C2—C3	1.446 (3)	C5—H5	0.930
C2—C11	1.367 (2)	C6—H6	0.930
C3—C4	1.415 (3)	C7—H7	0.930
C3—C8	1.424 (2)	C9—H9	0.930
C4—C5	1.373 (3)	C10—H10	0.930
C5—C6	1.397 (3)	C13—H13	0.930
C6—C7	1.356 (4)	C15—H15	0.930
C7—C8	1.416 (3)	C16—H16	0.930
C8—C9	1.407 (3)	C18—H18	0.930
C9—C10	1.353 (3)	C19—H19	0.930
C10—C11	1.411 (2)	C21—H21	0.930
C12—C13	1.308 (2)	C22—H22	0.930
C12—C14	1.484 (3)	C24—H24	0.930
C14—C15	1.381 (3)	C25—H25	0.930
C14—C19	1.383 (2)	C26—H261	0.960
C15—C16	1.391 (3)	C26—H262	0.960
C16—C17	1.368 (3)	C26—H263	0.960
C17—C18	1.372 (3)		
			
C11—O1—C12	117.40 (15)	C21—C22—C23	119.3 (2)
C2—C1—C13	108.24 (16)	Cl2—C23—C22	120.80 (18)
C2—C1—C20	109.59 (17)	Cl2—C23—C24	118.6 (2)
C2—C1—C26	111.80 (18)	C22—C23—C24	120.6 (2)
C13—C1—C20	106.20 (17)	C23—C24—C25	119.3 (2)
C13—C1—C26	106.86 (18)	C20—C25—C24	122.2 (2)
C20—C1—C26	113.82 (17)	C3—C4—H4	119.0
C1—C2—C3	122.33 (17)	C5—C4—H4	119.0
C1—C2—C11	120.91 (18)	C4—C5—H5	119.8
C3—C2—C11	116.76 (17)	C6—C5—H5	119.8
C2—C3—C4	123.51 (18)	C5—C6—H6	120.1
C2—C3—C8	119.56 (19)	C7—C6—H6	120.1
C4—C3—C8	116.9 (2)	C6—C7—H7	119.3
C3—C4—C5	121.9 (2)	C8—C7—H7	119.3
C4—C5—C6	120.3 (2)	C8—C9—H9	119.5
C5—C6—C7	119.8 (2)	C10—C9—H9	119.5
C6—C7—C8	121.4 (2)	C9—C10—H10	120.4
C3—C8—C7	119.6 (2)	C11—C10—H10	120.4
C3—C8—C9	119.6 (2)	C1—C13—H13	116.9
C7—C8—C9	120.82 (19)	C12—C13—H13	116.9
C8—C9—C10	120.91 (19)	C14—C15—H15	119.6
C9—C10—C11	119.3 (2)	C16—C15—H15	119.6
O1—C11—C2	124.47 (17)	C15—C16—H16	120.5
O1—C11—C10	111.76 (17)	C17—C16—H16	120.5
C2—C11—C10	123.8 (2)	C17—C18—H18	120.6
O1—C12—C13	122.56 (19)	C19—C18—H18	120.6
O1—C12—C14	110.38 (16)	C14—C19—H19	119.3
C13—C12—C14	127.05 (18)	C18—C19—H19	119.3
C1—C13—C12	126.12 (19)	C20—C21—H21	119.3
C12—C14—C15	120.82 (19)	C22—C21—H21	119.3
C12—C14—C19	120.67 (19)	C21—C22—H22	120.4
C15—C14—C19	118.5 (2)	C23—C22—H22	120.4
C14—C15—C16	120.7 (2)	C23—C24—H24	120.4
C15—C16—C17	119.1 (2)	C25—C24—H24	120.4
Cl1—C17—C16	119.3 (2)	C20—C25—H25	118.9
Cl1—C17—C18	119.22 (18)	C24—C25—H25	118.9
C16—C17—C18	121.5 (2)	C1—C26—H261	109.5
C17—C18—C19	118.9 (2)	C1—C26—H262	109.5
C14—C19—C18	121.4 (2)	C1—C26—H263	109.5
C1—C20—C21	124.8 (2)	H261—C26—H262	109.5
C1—C20—C25	118.00 (17)	H261—C26—H263	109.5
C21—C20—C25	117.2 (2)	H262—C26—H263	109.5
C20—C21—C22	121.4 (2)		
			
C11—O1—C12—C13	3.5 (3)	C5—C6—C7—C8	−0.5 (4)
C11—O1—C12—C14	−177.32 (19)	C6—C7—C8—C3	−0.9 (3)
C12—O1—C11—C2	−6.0 (3)	C6—C7—C8—C9	179.0 (2)
C12—O1—C11—C10	173.84 (19)	C3—C8—C9—C10	2.0 (3)
C2—C1—C13—C12	−3.8 (3)	C7—C8—C9—C10	−177.9 (2)
C13—C1—C2—C3	−179.1 (2)	C8—C9—C10—C11	−1.7 (3)
C13—C1—C2—C11	1.3 (2)	C9—C10—C11—O1	178.7 (2)
C2—C1—C20—C21	−131.0 (2)	C9—C10—C11—C2	−1.5 (3)
C2—C1—C20—C25	50.4 (2)	O1—C12—C13—C1	1.6 (3)
C20—C1—C2—C3	65.5 (2)	O1—C12—C14—C15	133.8 (2)
C20—C1—C2—C11	−114.1 (2)	O1—C12—C14—C19	−45.6 (2)
C26—C1—C2—C3	−61.6 (2)	C13—C12—C14—C15	−47.0 (3)
C26—C1—C2—C11	118.8 (2)	C13—C12—C14—C19	133.5 (2)
C13—C1—C20—C21	112.3 (2)	C14—C12—C13—C1	−177.5 (2)
C13—C1—C20—C25	−66.3 (2)	C12—C14—C15—C16	−179.0 (2)
C20—C1—C13—C12	113.8 (2)	C12—C14—C19—C18	179.9 (2)
C26—C1—C13—C12	−124.4 (2)	C15—C14—C19—C18	0.4 (3)
C26—C1—C20—C21	−5.0 (3)	C19—C14—C15—C16	0.4 (3)
C26—C1—C20—C25	176.4 (2)	C14—C15—C16—C17	−0.6 (4)
C1—C2—C3—C4	−4.6 (3)	C15—C16—C17—Cl1	−179.6 (2)
C1—C2—C3—C8	176.9 (2)	C15—C16—C17—C18	−0.0 (3)
C1—C2—C11—O1	3.4 (3)	Cl1—C17—C18—C19	−179.58 (19)
C1—C2—C11—C10	−176.4 (2)	C16—C17—C18—C19	0.8 (3)
C3—C2—C11—O1	−176.2 (2)	C17—C18—C19—C14	−1.0 (3)
C3—C2—C11—C10	4.0 (3)	C1—C20—C21—C22	−179.8 (2)
C11—C2—C3—C4	175.1 (2)	C1—C20—C25—C24	−179.5 (2)
C11—C2—C3—C8	−3.4 (3)	C21—C20—C25—C24	1.8 (3)
C2—C3—C4—C5	179.8 (2)	C25—C20—C21—C22	−1.2 (3)
C2—C3—C8—C7	−179.5 (2)	C20—C21—C22—C23	−0.6 (3)
C2—C3—C8—C9	0.6 (3)	C21—C22—C23—Cl2	−177.82 (19)
C4—C3—C8—C7	1.9 (3)	C21—C22—C23—C24	1.9 (3)
C4—C3—C8—C9	−178.0 (2)	Cl2—C23—C24—C25	178.40 (19)
C8—C3—C4—C5	−1.7 (3)	C22—C23—C24—C25	−1.3 (3)
C3—C4—C5—C6	0.4 (4)	C23—C24—C25—C20	−0.6 (3)
C4—C5—C6—C7	0.7 (4)		
